# Predicting placenta accreta spectrum antenatally: a deep learning-radiomics dual-model based on intraoperative clinical and histopathologic criteria

**DOI:** 10.1186/s12880-026-02538-2

**Published:** 2026-07-17

**Authors:** Yumin Hu, Chenqi Shen, Di Shen, Xiaojun Chen, Hongyan Zhao, Xia Li, Qing Zhou, Feng Shi, Yechao Huang, Wensong Xu, Chenying Lu

**Affiliations:** 1 Department of Radiology, The Fifth Affiliated Hospital of Wenzhou Medical University, Lishui, 323000 China; 2 Department of Radiology, The Second Affiliated Hospital and Yuying Children’s Hospital, Wenzhou Medical University, Wenzhou, China; 3 Department of Obstetrics, The Fifth Affiliated Hospital of Wenzhou Medical University, Lishui, China; 4 Department of Research and Development, Shanghai United Imaging Intelligence Co., Ltd, Shanghai, Shanghai China; 5 Hangzhou Medical College, Hangzhou, China

**Keywords:** Placenta accreta spectrum, Deep learning, Histopathologic criteria, Intraoperative clinical criteria, Radiomics

## Abstract

**Purpose:**

Definitive diagnosis of placenta accreta spectrum (PAS) disorders occurs at delivery by using combined intraoperative clinical and histopathologic diagnostic criteria per the recently updated International Federation of Gynecology and Obstetrics (FIGO) classification system. The purpose of this study is to construct a dual diagnostic model based on intraoperative clinical and histopathologic criteria for PAS in accordance with FIGO’s diagnostic criteria.

**Materials and methods:**

This study pooled data from two centers. Diagnostic models based on histopathologic and intraoperative clinical criteria were separately developed using radiomics, deep learning, and clinical features. Subsequently, these individual models were integrated. Moreover, the correlations among clinical indicators, radiomics, and deep-learning features were explored. The Mann-Whitney U test was used for continuous variables with non-normal distribution. Categorical data were analyzed by Fisher’s exact test or 2 × 2 or R×C χ2 test. A P value < 0.05 was considered significant.

**Results:**

For the FIGO intraoperative clinical criteria model, the fusion model’s AUC was 0.964 (0.946–0.982) in the training set and 0.949 (0.902–0.996) in the test set. For the FIGO histopathologic criteria model, the training-set AUC was 0.961 (0.940–0.982), and the validation cohort AUC was 0.936 (0.883–0.990). In addition, several clinical factors and magnetic resonance imaging (MRI) features of PAS were found to correlate with radiomics scores and deep learning scores in both the intraoperative clinical and histopathologic criteria models.

**Conclusion:**

In this study, we constructed a dual model that includes FIGO intraoperative clinical and histopathologic criteria for predicting PAS. The two models that integrate deep learning, radiomics, and clinical features have good performance.

**Supplementary Information:**

The online version contains supplementary material available at https://doi.org/10.1186/s12880-026-02538-2.

## Introduction

Placenta accreta spectrum (PAS) disorders are a major cause of maternal morbidity and mortality and are increasing in incidence owing to a rising rate of repeat cesarean sections. The incidence of PAS disorders has increased approximately 10-fold in the past 50 years [1, 2]. Definitive diagnosis of PAS disorders is made at delivery using combined intraoperative clinical and histopathologic criteria per the recently updated International Federation of Gynecology and Obstetrics (FIGO) classification system. The FIGO classification system comprises histopathologic and intraoperative clinical grading criteria [3–8]. Intraoperative clinical diagnostic criteria are determined by obstetricians based on intraoperative findings, whereas histopathologic diagnostic criteria are established based on postoperative surgical specimens [3, 9]. Both FIGO grading systems (intraoperative clinical and histopathologic) are equally crucial for PAS management. In clinical practice, the intraoperative clinical and histopathologic grading diagnoses of the same PAS patient may be inconsistent, and significant discrepancies may even exist between the two. Histopathologic grading criteria are indeed critical for determining the depth of placental invasion into the myometrium. While histopathologic grading is important for determining placental invasion depth, it has limitations: limited value in managing retained placenta [10], potential underestimation due to inadequate intraoperative sampling of the uteroplacental interface, and a lack of data from patients with mild but extensive invasion who do not undergo hysterectomy [6]. In response, FIGO proposed an alternative intraoperative clinical classification for placental adherence at delivery [3], but core diagnostic challenges persist.

Accurate antenatal diagnosis and multidisciplinary management are critical for guiding treatment and ensuring optimal outcomes in PAS patients [11]. Ultrasound (US) and magnetic resonance imaging (MRI) are the current imaging modalities of choice for detecting PAS, as both employ noninvasive and nonionizing diagnostic techniques [12]. However, as has emerged from recent studies, the diagnostic accuracy of PAS detection using US and MRI largely depends on the extensive experience of sonographers and radiologists [13, 14]. AI-based medical imaging methods primarily include radiomics and deep learning. Currently, some studies have applied radiomics to the diagnosis of placental accreta spectrum diseases [15–23]. However, it remains unclear whether intraoperative clinical or histopathologic criteria were used to define PAS in many of these studies [24]. Many studies have combined intraoperative clinical and histopathologic diagnoses in a single study, while others focus solely on histopathologic diagnosis. In addition, there is not much research on the application of deep learning and radiomics comparison and fusion in predicting PAS.

In our study, we aimed to construct a dual model incorporating FIGO intraoperative clinical and histopathologic criteria for predicting PAS. We primarily applied AI methods, including radiomics and deep learning, and incorporated both MRI imaging features and clinical data.

## Materials and methods

### Pregnant women

This retrospective study was approved by the Institutional Review Boards (IRBs) of both centers with a waiver of informed consent (Approval No.2026(I)-052-01). Placental MRI and clinical data were collected from pregnancies suspected of PAS before cesarean section at Institution 1 (*n* = 215; September 2019-December 2023) and Institution 2 (*n* = 200; January 2019-March 2024).

**Fig. 1:**
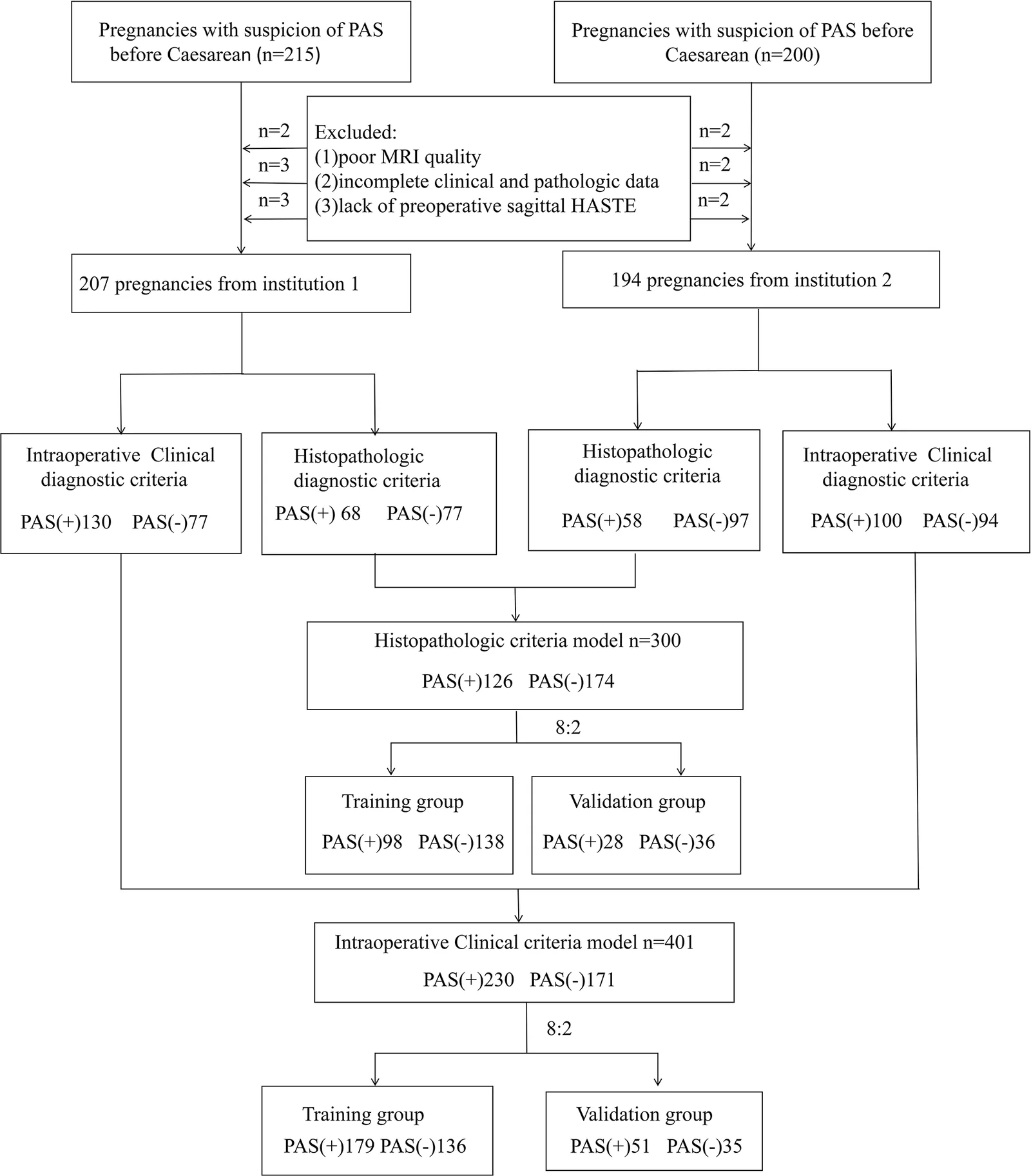
The flowchart illustrates the participant enrollment pathway, including the distribution of pregnant women into the training and validation cohorts for both the intraoperative clinical and histopathological criteria models

Inclusion criteria: (1) Singleton pregnancies with suspected PAS disorders who underwent prenatal MRI for placental assessment before cesarean delivery; (2) MRI scans were of diagnostic quality; (3) Complete clinical and pathological data. Exclusion criteria: (1) Inadequate image quality; (2) Incomplete clinical and pathological data; (3) Lack of preoperative sagittal HASTE sequences. Ultimately, 207 pregnancies from Institution 1 and 194 from Institution 2 were included in the study. The diagnostic criteria for PAS are key bases for grouping in this study. All intraoperative clinical and pathological diagnoses of PAS in this study were in accordance with FIGO’s intraoperative clinical and histopathologic diagnostic criteria. According to FIGO intraoperative clinical diagnostic criteria, the 207 cases from Institution 1 were categorized into 130 PAS-positive cases and 77 PAS-negative cases. Similarly, the 194 cases from Institution 2 were categorized into 100 PAS-positive and 94 PAS-negative cases. Concurrently, based on FIGO histopathologic diagnostic criteria, 68 PAS-positive and 77 PAS-negative cases were reselected from Institution 1, and 58 PAS-positive and 97 PAS-negative cases from Institution 2. To increase the sample size and develop a more stable model, cases from both institutions were combined. The 101-case discrepancy represents cases clinically diagnosed with placenta accreta intraoperatively but not confirmed by final histopathology which based on the FIGO intraoperative clinical and histopathologic diagnostic criteria [6]. The 101 discrepant cases (intraoperative PAS-positive but histopathology-negative) were included to construct intraoperative clinical model. We fully recognize that these cases are often accompanied by critical conditions such as severe intraoperative hemorrhage. This is exactly why we established two independent models based on separate diagnostic criteria. Our definitive PAS-negative control group included only cases negative for placenta accreta based on both intraoperative clinical and histopathologic criteria. This rigorous differentiation underscores the significance of our study. For constructing the dual model, pregnant women were randomly divided into training and validation cohorts at an 8:2 ratio for both the intraoperative clinical and histopathologic criteria models. The flowchart of the enrollment pathway and study design is shown in Fig. 1. Previously, we developed a radiomic model for PAS using MRI images of 132 pregnant women suspected of the disease, which integrated both pathological and intraoperative clinical diagnoses of PAS [25]. This model integrates pathological diagnosis and intraoperative clinical diagnosis of PAS into one model. Clinical practice has shown that developing separate models is more clinically relevant. Thus, we enrolled additional pregnant women to develop separate models.

### MR image acquisition

Placental MRI images for all pregnant women were acquired using a 1.5-T MRI scanner (MAGENTOM Area; Siemens) at Institution 1 and a 1.5-T MRI scanner (MAGENTOM Avanto; Siemens) at Institution 2. Both institutions employed 18-channel body array coils for signal reception. The sequence used in this study was sagittal Fourier acquisition single-shot turbo spin-echo (HASTE). MRI sequence parameters for both institutions are provided in Supplementary Table S1.

### MRI morphologic assessment of PAS

Two radiologists with 16 and 21 years of diagnostic imaging experience independently evaluated the imaging findings using the RAD-info RSVS system. Both readers were blinded to prior MRI and ultrasound reports, as well as intraoperative and postoperative pathological reports. Any discrepancies were resolved by consensus.This study, based on the joint statement by the European Society of Urogenital Radiology (ESUR) and the Society of Abdominal Radiology (SAR), documented five key MRI features: intraplacental dark T2 bands (IDTB), placental bulge, myometrial thinning/disruption, increased placental vascularity (IPV), and bladder wall interruption [26]. Additionally, based on previous studies, the number of IDTBs, as well as the long diameter (LD), short diameter, and area of the largest intraplacental dark T2 band (LIDTB), were included [27]. Furthermore, the location of IPV and vessel diameter were also included [28]. Cases with a history of cesarean section and a placenta located on the anterior lower uterine wall were specifically documented. The maximum placental thickness was also documented.

### Placental tissue segmentation

Automatic segmentation was used to segment placental tissue. Sagittal HASTE sequences were used for both uterine/placental evaluation and placental tissue segmentation. Following automatic segmentation, a radiologist with 12 years of experience verified the placental segmentation and manually delineated any incorrectly segmented sections. This primarily involved removing surrounding decidual structures outside the placental range and delineating any undelineated placental tissue. All ROIs were subject to an independent review conducted by a second radiologist (with > 16 years of experience). If two confirmed radiologists have different opinions on the delineation range of the placenta, they shall jointly discuss and determine it.

### Radiomics, deep learning and clinical model development

Confusion matrices for classifying PAS-positive and PAS-negative cases using the clinical, radiomics, DenseNet, ResNet, and fusion models are presented in Supplementary Figure S1 for both cohorts, selected based on established histopathologic and intraoperative clinical criteria.

The Radiomic features were extracted from segmented ROIs analyzed using the PyRadiomics package (version 3.0.1; http://www.radiomics.io/pyradiomics.html) and uAI Research Portal (Shanghai United Imaging Intelligence Co., Ltd., Version: 20231115) [29]. Most radiomic features were defined in accordance with the Image Biomarkers Standardization Initiative (IBSI). In both the histopathologic and intraoperative clinical criteria diagnostic models, a comprehensive set of radiomics features was ultimately extracted from each image. These encompassed first-order (18), shape (14), and texture (72) features.

The optimal penalty coefficient (λ = 3.39 × 10⁻²) was determined using LASSO regression with 10,000 iterations of ten-fold cross-validation. This process selected 21 features with non-zero coefficients, which were then input into a support vector machine (SVM) to develop the radiomic model (see Appendix E1).

**Fig. 2:**
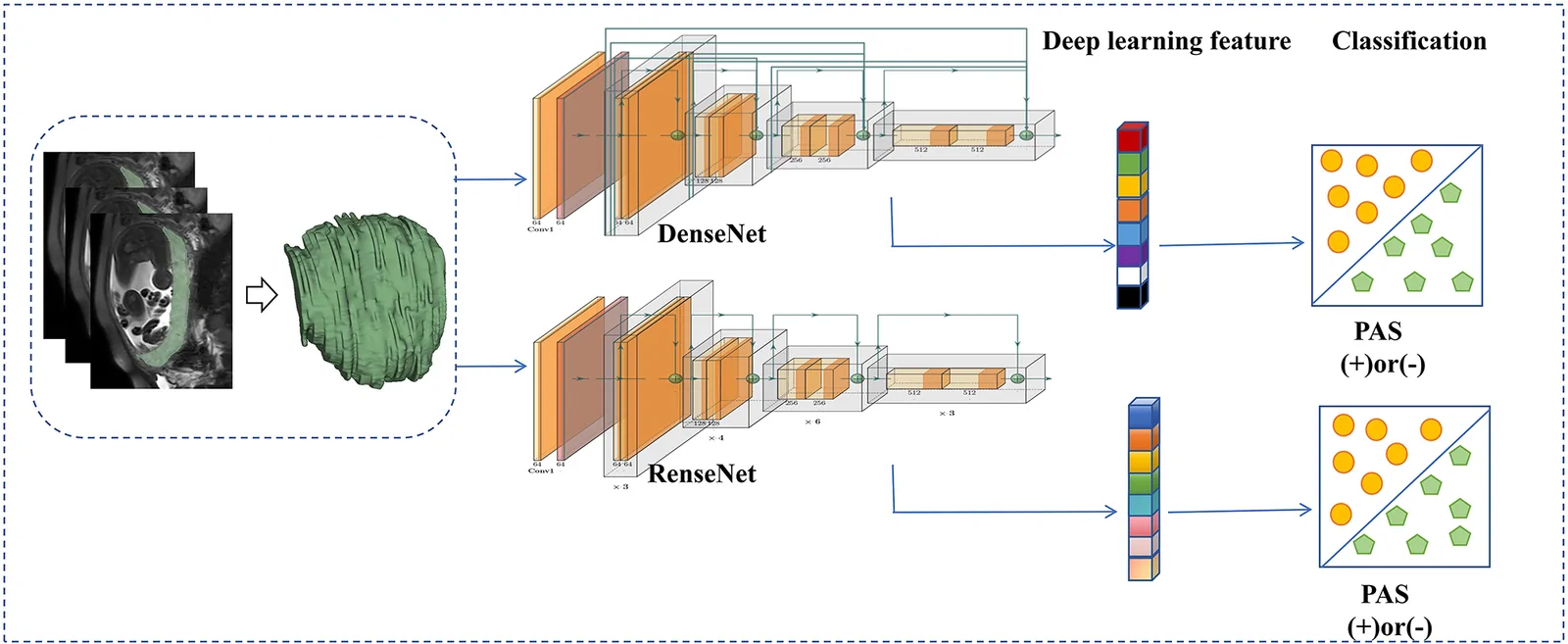
The schematic depicts our 3D DenseNet and ResNet ensemble, wherein input images undergo successive convolution and pooling operations to differentiate between PAS and non-PAS cases. The output of each layer in ResNet is combined with the input from the previous layer, expressed as follows: Xl = Hl(Xl-1) + Xl-1.And DenseNet will superimpose the outputs of all previous feature layers at each layer and pass them on to the next layer, that is, that is, Xl = Hl ([X0, X1,…, Xl-1])

To capture the most discriminative placental tissue features, deep learning models with more effective feature extraction capabilities were constructed. DenseNet, a type of convolutional neural network (CNN), comprises four dense blocks, each characterized by dense interlayer connections. DenseNet was selected as the base model for this study due to its unique advantages. ResNet, in contrast, excels at utilizing automatic parameter learning to extract contextual features while preserving three-dimensional information. This property makes it a valuable choice for deep learning tasks similar to ours. Together, these models provide distinct feature extraction approaches, which we believe will significantly enhance our understanding of placental tissue features and ultimately yield more accurate and insightful results (see Appendix E2). The overall workflow is outlined in Fig. 2.

Univariate and multivariate binary logistic regression analyses were performed to identify the most influential and independent clinical factors significantly associated with the outcome. Clinical indicators with a P-value < 0.05 in multivariate binary logistic regression were included in the clinical model development.

### The multi-modality fusion model development and comparison

A multimodal fusion model was constructed by integrating the clinical, radiomic, and deep learning models to evaluate their combined value. To generate a composite score, the outputs of all three models were integrated for each participant using multivariate logistic regression. The combined model’s output was converted into a predicted probability (ranging from 0 to 1) using the sigmoid function of logistic regression. The interpretable weights of logistic regression allow for assessing the relative influence of each individual model on the combined output. Its applicability to binary outcomes facilitates clinicians’ understanding and adoption of the model. The model was designed to output both a binary diagnosis and a probabilistic score, thereby enhancing clinicians’ trust and understanding.

We compared the performance of the combined model with the three individual models, employing discriminatory power (AUC) and its classification performance (sensitivity, specificity, and accuracy) as metrics. The calibration curve assessed the predictive performance of the combined model. Additionally, decision curve analysis (DCA) was used to quantify its clinical utility by estimating the net benefit across a range of threshold probabilities. To evaluate the predictive ability and stability of the model, the dataset was randomly split into training and validation cohorts at an 8:2 ratio. Furthermore, we compared the predictions of the four models for two representative cases (one PAS-positive and one PAS-negative).

### Construction of the nomogram based on the fusion model

Following careful screening of clinical factors, the clinical indicators derived from multivariate binary logistic regression were combined with three distinct scores: the Rad-score, ResNet-score, and DenseNet-score. The Rad-score was calculated based on radiomic features, which encapsulate a broad range of quantitative information from medical images. The ResNet-score and DenseNet-score, in contrast, were derived from deep features extracted from the ResNet and DenseNet architectures, respectively. These scores represent the complex, high-level features learned by the neural networks.

**Fig. 3:**
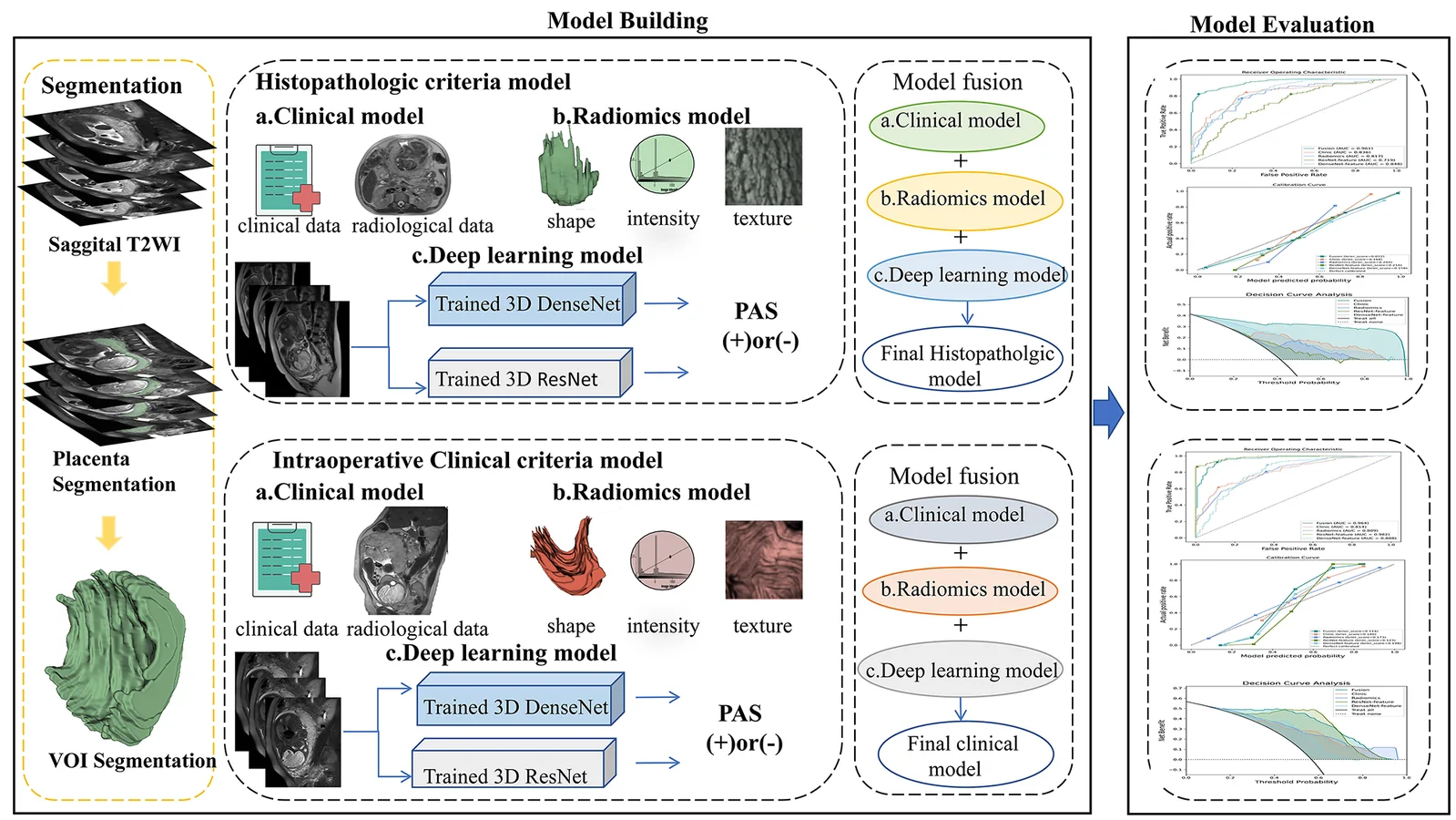
The study workflow comprised the following steps: (1) Segmentation of the Regions of Interest (ROI); (2) Construction of intraoperative clinical and histopathological criteria models, each encompassing a clinical model, a radiomics model, and a deep learning model; (3) Fusion of the individual models; (4) Model evaluation using the area under the receiver operating characteristic curve (AUC), calibration curves, and decision curve analysis to assess predictive performance and clinical utility

By integrating these diverse data types, a nomogram—a graphical tool that combines multiple variables into a single, user-friendly format—was developed. It provides a visual representation of the relationship between the input variables (clinical indicators, Rad-score, ResNet-score, and DenseNet-score) and the predicted outcome [30]. This nomogram can serve as a valuable tool in clinical decision-making, enabling healthcare professionals to rapidly and accurately estimate the probability of a specific outcome based on a patient’s individual characteristics. The overall research workflow is summarized in Fig. 3.

### Correlation between radiomic/deep learning features and clinical indicators

Pearson correlation analysis was performed to assess the relationships between clinical indicators derived from multivariate logistic regression, radiomic scores, and deep learning scores (ResNet and DenseNet). To effectively visualize and interpret the complex relationships among these variables, an interaction network correlation pie chart was used.

### Statistical analysis

For baseline demographic and clinical characteristics, the Mann-Whitney U test was used for continuous variables with non-normal distribution. Categorical data were analyzed using Fisher’s exact test, 2 × 2 χ² test, or R×C χ² test. Statistical analyses were performed using Python 3.10.6. All statistical tests were two-sided, and P-values < 0.05 were considered statistically significant. Missing values were imputed with the median of the respective variable. Screening of clinical features for nomogram construction was performed using binary univariate and multivariate regression in SPSS. Statistical analysis and visualization of the relevant string plots were performed using R version 4.2.1. The R packages involved were igraph [1.3.4] and ggraph [2.1.0].

## Results

### Clinical and radiological characteristics of pregnant women in the intraoperative clinical and histopathologic criteria models

Table 1 provides a summary of the clinical characteristics of all pregnancies in the training and validation cohorts of **intraoperative** clinical and histopathological criteria models. In both the **intraoperative** clinical and histopathologic criteria models, statistically significant differences were observed between the PAS-positive and PAS-negative groups in the training cohort for gestational age at cesarean delivery (CD) (both *P* < 0.001), number of CDs (both *P* < 0.001), type of placenta previa (*P* = 0.005 and *P* = 0.006), number of vaginal deliveries (VD) (*P* = 0.012 and *P* = 0.002), number of abortions (*P* = 0.001 and *P* = 0.037), and preoperative fibrinogen level (both *P* < 0.001). In the validation cohort of the intraoperative clinical criteria model, statistically significant differences were noted in maternal age (*P* = 0.030), number of abortions (*P* = 0.007), preoperative activated partial thromboplastin time (APTT) (*P* = 0.034), and fibrinogen level (*P* = 0.015). In the validation cohort of the histopathologic criteria model, statistically significant differences were observed in maternal age (*P* = 0.031), gestational age at CD (*P* = 0.050), number of abortions (*P* = 0.048), antenatal hemorrhage (*P* = 0.034), preoperative platelet count (*P* = 0.008), and APTT level (*P* = 0.020).

**Table 1:** Clinical characteristics of patients in Intraoperative Clinical and Histopathologic criteria model

Dual model			Intraoperative Clinical criteria model							Histopathologic criteria model						
			Training cohort			Testing cohort			*p*-inter	Training cohort			Testing cohort			*p*-inter
Characteristics			PAS- (*n* = 136)	PAS+ (*n* = 179)	p-intra	PAS- (*n* = 35)	PAS+ (*n* = 51)	p-intra		PAS- (*n* = 138)	PAS+ (*n* = 98)	p-intra	PAS- (*n* = 36)	PAS+ (*n* = 28)	p-intra	
Maternal age, years			32.00 (29.00,36.00)	33.00 (30.00,37.00)	0.266	33.00 (30.50,35.00)	36.00 (32.00,39.00)	0.030	0.047	32.000 (29.00,36.00)	33.00 (30.00,37.00)	0.213	33.00 (30.75,35.00)	36.00 (33.00,39.00)	0.031	0.060
Gestational age at MRI, weeks			34.00 (32.00,35.00)	34.00 (32.00,35.00)	0.525	33.000 (32.00,34.00)	33.000 (32.00,34.00)	0.759	0.119	34.000 (32.00,35.00)	34.000 (33.00,34.75)	0.554	33.000 (32.00,34.00)	32.500 (31.00,34.00)	0.373	0.01
Gestational age at CD , weeks			37.00 (36.00,37.00)	36.000 (35.00,37.00)	< 0.001	36.000 (36.00,37.00)	36.000 (35.00,37.00)	0.353	0.592	37.00 (36.00,37.00)	36.000 (35.00,37.00)	< 0.001	36.000 (36.00,37.00)	36.00 (34.75,36.00)	0.050	0.123
Number of CD (%)	0 1 2 3 4		84 (61.765) 49 (36.029) 3 (2.206) 0 (0.000) 0 (0.000)	83 (46.369) 70 (39.106) 24 (13.408) 1 (0.559) 1 (0.559)	< 0.001	23 (65.714) 11 (31.429) 1 (2.857) 0 (0.000) 0 (0.000)	26 (50.980) 22 (43.137) 3 (5.882) 0 (0.000) 0 (0.000)	0.449	0.695	86 (62.319) 49 (35.507) 3 (2.174) 0 (0.000)	43 (43.878) 39 (39.796) 15 (15.306) 1 (1.020)	< 0.001	23 (63.889) 12 (33.333) 1 (2.778) 0 (0.000)	14 (50.000) 11 (39.286) 3 (10.714) 0 (0.000)	0.362	0.943
Number of VD (%)	0 1 2 3 4		82 (60.294) 46 (33.824) 7 (5.147) 1 (0.735) 0 (0.000)	136 (75.978) 34 (18.994) 6 (3.352) 2 (1.117) 1 (0.559)	0.012	21 (60.000) 14 (40.000) 0 (0.000) 0 (0.000) 0 (0.000)	34 (66.667) 14 (27.451) 2 (3.922) 1 (1.961) 0 (0.000)	0.419	0.610	83 (60.145) 47 (34.058) 7 (5.072) 1 (0.725) 0 (0.000)	79 (80.612) 16 (16.327) 2 (2.041) 0 (0.000) 1 (1.020)	0.002	22 (61.111) 14 (38.889) 0 (0.000) 0 (0.000) 0 (0.000)	19 (67.857) 8 (28.571) 1 (3.571) 0 (0.000) 0 (0.000)	0.424	0.653
Number of abortion			1.000 (0.00,2.000)	1.000 (1.00,2.00)	0.010	1.000 (0.00,2.00)	2.000 (1.00,3.00)	0.007	0.116	1.000 (0.000,2.000)	1.000 (1.000,2.000)	0.037	1.000 (0.000,2.000)	2.000 (1.000,3.000)	0.048	0.361
Placenta previa (%)	- +		6 (4.412) 130 (95.588)	5 (2.793) 174 (97.207)	0.438	2 (5.714) 33 (94.286)	2 (3.922) 49 (96.078)	0.698	0.616	6 (4.348) 132 (95.652)	2 (2.041) 96 (97.959)	0.335	2 (5.556) 34 (94.444)	1 (3.571) 27 (96.429)	0.709	0.624
Type of placenta previa (%)	**None** **Complete** **Partial** **Marginal** **Low‑lying**		**6 (4.412)** **90 (66.176)** 8 (5.882) 17 (12.500) 15 (11.029)	5 (2.793) 149 (83.240) 6 (3.352) 14 (7.821) 5 (2.793)	0.005	2 (5.714) 22 (62.857) 2 (5.714) 4 (11.429) 5 (14.286)	2 (3.922) 40 (78.431) 1 (1.961) 4 (7.843) 4 (7.843)	0.569	0.690	6 (4.348) 91 (65.942) 8 (5.797) 18 (13.043) 15 (10.870)	2 (2.041) 85 (86.735) 1 (1.020) 7 (7.143) 3 (3.061)	0.006	2 (5.556) 23 (63.889) 2 (5.556) 4 (11.111) 5 (13.889)	1 (3.571) 23 (82.143) 0 (0.000) 2 (7.143) 2 (7.143)	0.602	0.862
Number of antenatal hemorrhage			0.000 (0.00,1.00)	1.00 (0.00,2.00)	0.064	0.000 (0.00,1.50)	1.000 (0.00,2.00)	0.158	0.184	0.000 (0.000,1.000)	1.000 (0.000,2.000)	0.160	0.000 (0.000,2.000)	1.000 (0.000,2.250)	0.034	0.032
Postpartum Hemorrhage		<1000 ml ≥ 1000 ml	PAS(-)			PAS(+)			<0.001	PAS(-)			PAS(+)			<0.001
			160 11			142 91				161 13			74 53			
Surgical approach		TH SH FR SPS	PAS(-)			PAS(+)			<0.001	PAS(-)			PAS(+)			<0.001
			0(0.000) 0(0.000) 0(0.000) 171(1.000)			9(0.039) 30(0.130) 191(0.830) 0(0.000)				0(0.000) 0(0.000) 20(0.115) 154(0.885)			9(0.071) 20(0.159) 97(0.770) 0(0.000)			
Preoperative hemoglobin level ^†^			114.500 (107.750,123.000)	113.000 (103.500,120.000)	0.046	116.000 (111.000,121.500)	116.000 (104.000,125.000)	0.460	0.220	114.000 (107.250,123.000)	113.000 (102.250,120.000)	0.080	116.000 (109.750,121.250)	112.500 (103.750,118.250)	0.083	0.913
Preoperative platelet level			200.000 (162.750,235.250)	199.000 (159.500,235.000)	0.975	195.000 (164.000,231.500)	211.000 (179.500,253.500)	0.112	0.165	202.500 (163.750,239.000)	201.500 (152.250,231.750)	0.577	201.500 (165.500,233.000)	243.500 (195.500,285.250)	0.008	0.053
Preoperative INR			0.960 (0.930,1.000)	0.970 (0.930,1.030)	0.225	0.980 (0.940,1.010)	0.980 (0.920,1.020)	0.689	0.618	0.960 (0.930,1.000)	0.970 (0.930,1.020)	0.320	0.980 (0.940,1.012)	0.975 (0.938,1.012)	0.597	0.389
Preoperative APTT			30.450 (27.00,32.80)	28.800 (26.05,32.35)	0.083	29.800 (27.25,33.00)	28.200 (25.75,31.15)	0.034	0.522	30.600 (27.075,32.750)	29.500 (26.725,32.675)	0.465	30.200 (27.275,32.750)	27.400 (26.100,29.750)	0.020	0.32
Preoperative D2			1.600 (1.470,1.762)	1.600 [1.315,2.210]	0.36	1.600 (1.325,1.600)	1.600 (1.095,2.150)	0.828	0.313	1.650 (1.472,1.748)	1.650 (1.425,2.362)	0.055	1.650 (1.302,1.650)	1.650 (1.098,2.252)	0.742	0.176
Preoperative fibrinogen			4.480 (4.010,5.000)	4.110 (3.560,4.745)	< 0.001	4.590 (3.925,5.060)	4.180 (3.550,4.660)	0.015	0.692	4.480 (4.010,5.000)	4.000 (3.527,4.633)	< 0.001	4.595 (3.938,5.060)	4.210 (3.683,4.803)	0.144	0.532

Abbreviations: CD=Cesarean Delivery, VD=Vaginal delivery, INR=International Normalized R Ratio, APTT=Activated Partial Thromboplastin Time, D2 = D-Dimer, TH=Total Hysterectomy, SH= Subtotal Hysterectomy, FR=Focal resection, SPS=Spontaneous Placental Separation

Supplementary Table S2 provides a summary of the radiological characteristics of all pregnancies from the training and validation cohorts of the intraoperative clinical and histopathologic criteria models. MRI features related to IDTB, IPV, and myometrial thinning/disruption showed statistically significant differences between PAS-positive and PAS-negative cases in each cohort (all *P* < 0.001). Placental bulge showed statistically significant differences in the training cohorts of the intraoperative clinical (*P* = 0.001) and histopathologic criteria models (*P* < 0.001). Placental thickness and placental coverage of the uterine scar showed statistically significant differences between PAS-positive and PAS-negative cases in the training cohorts of the intraoperative clinical (*P* = 0.012 and *P* < 0.001) and histopathologic criteria models (*P* = 0.015 and *P* < 0.001), as well as in the validation cohort of the histopathologic criteria model (*P* = 0.030 and *P* = 0.017).

### Construction of a dual model for intraoperative clinical and histopathologic criteria-based diagnosis of PAS

#### Intraoperative clinical criteria model construction

##### Radiomics model

**Fig. 4:**
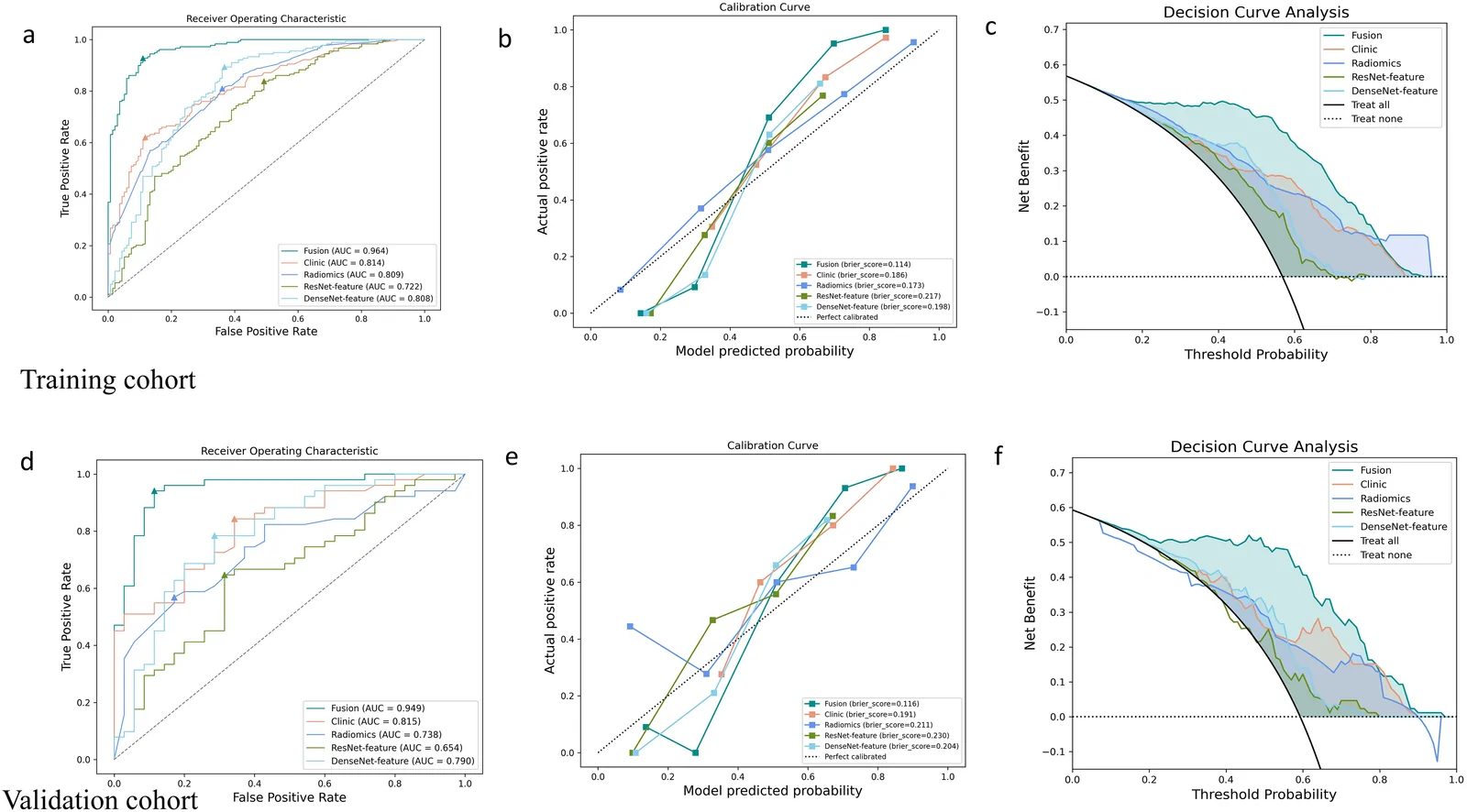
**a**. ROC curves of intraoperative clinical criteria model, including radiomics model, DenseNet deep learning model, ResNet deep learning model and fusion model in training cohort. **b**. Calibration curve of different models in training cohort. **c**. decision curve of different models in training cohort. **d**. ROC curves of intraoperative clinical criteria model, including radiomics model, DenseNet deep learning model, ResNet deep learning model and fusion model in validation cohort. **e**. decision curve of different models in validation cohort. **f**. decision curve of different models in validation cohort

The radiomic model incorporating 29 selected features achieved an AUC of 0.809 (95% CI: 0.762, 0.873) in the training cohort and 0.738 (95% CI: 0.633, 0.844) in the validation cohort. The accuracy (ACC), sensitivity (SN), and specificity (SP) were 0.727, 0.782, and 0.654 in the training cohort and 0.698, 0.745 and 0.629 in the validation cohort, respectively (Table 2; Figs. 4a, d).

**Table 2:** Performance of intraoperative clinical and histopathologic criteria models in training and validation cohorts

Intraoperative Clinical criteria model								
Machine learning model	Training cohort				Testing cohort			
	AUC	Sensitivity	Specificity	Accuracy	AUC	Sensitivity	Specificity	Accuracy
Clinical	0.814(0.767–0.860)	0.659	0.831	0.733	0.815(0.728–0.903)	0.569	0.800	0.663
Radiomics	0.809(0.762–0.873)	0.782	0.654	0.727	0.738(0.633–0.844)	0.745	0.629	0.698
ResNet feature	0.722(0.665–0.780)	0.0.710	0.610	0.667	0.654(0.536–0.772)	0.667	0.600	0.640
DenseNet feature	0.808(0.758–0.891)	0.777	0.699	0.743	0.79(0.689–0.891)	0.745	0.714	0.733
Fusion	0.964(0.946–0.982)	0.877	0.904	0.889	0.949(0.902–0.996)	0.941	0.886	0.919

**Histopathologic criteria model**								
**Machine learning model**	**Training cohort**				**Testing cohort**			
	**AUC**	**Sensitivity**	**Specificity**	**Accuracy**	**AUC**	**Sensitivity**	**Specificity**	**Accuracy**
Clinical	0.836(0.782–0.890)	0.684	0.804	0.754	0.8532(0.759–0.948)	0.857	0.722	0.781
Radiomics	0.817(0.762–0.872)	0.765	0.754	0.758	0.763(0.641–0.885)	0.714	0.722	0.719
ResNet feature	0.719(0.654–0.784)	0.694	0.63	0.657	0.68(0.539–0.82)	0.714	0.556	0.625
DenseNet feature	0.848(0.801–0.895)	0.898	0.616	0.733	0.765(0.648–0.882)	0.786	0.556	0.656
Fusion	0.961(0.940–0.982)	0.898	0.848	0.869	0.936(0.883–0.990)	0.821	0.861	0.844

Note. AUC = area under the curve

##### ResNet model

The AUC of ResNet in the training cohort was 0.722 (95% CI: 0.665,0.780), ACC of 0.667, SN of 0.710, and SP of 0.610, and the AUC in the validation cohort was 0.654 (95% CI: 0.536, 0.772), ACC of 0.640, SN of 0.667, and SP of 0.600 (Table 2; Figs. 4a, d).

##### DenseNet model

The AUC of DenseNet deep learning model for constructing intraoperative clinical criteria model was 0.808 (95% CI: 0.758,0.891) in the training cohort, 0.790 (95% CI: 0.689,0.891) in the validation cohort. ACC, SN, and SP values were 0.727, 0.782, 0.654 (training) and 0.698, 0.745, 0.629 (validation) (Table 2; Figs. 4a, d).

##### Clinical model

Eighteen clinical features were screened using univariate binary logistic regression for the clinical model (Table 3). The clinical model had an AUC of 0.814 (95% CI: 0.767, 0.860), SN of 0.659, SP of 0.831, and ACC of 0.733 in the training cohort, and an AUC of 0.815 (95% CI: 0.728, 0.903), SN of 0.569, SP of 0.800, and ACC of 0.663 in the validation cohort (Table 2; Figs. 4a, d).

**Table 3:** Results of the univariate and multivariate binary logistic regression analyses of histopathologic and intraoperative clinical criteria model

Intraoperative Clinical criteria model				
	Univariate logistic regression analyses		Multivariate logistic regression analyses	
	OR (95% CI)	*P*	OR (95% CI)	*P*
Gestational age at CD , weeks	0.601(0.461,0.784)	0.000	-	-
Number of VD	1.597(1.244,2.051)	0.000	-	-
Number of CD	0.791(0.630,0.993)	0.043	0.630(0.430,0.921)	0.017
Number of abortion	1.349(1.058,1.719)	0.016	1.454(1.209,1.750)	<0.001
Type of placenta previa	0.687(1.244,2.051)	0.001	-	-
Placenta cover uterus scar	1.972(1.515,2.566)	0.001	-	-
Preoperative fibrinogen	0.678(0.542,0.871)	0.002	0.620(0.473,0.814)	<0.001
IDTB	2.092(1.639,2.671)	0.000	-	-
Number of IDTB	2.940(2.020,4.279)	0.000	1.677(1.141,2.462)	0.008
Long diameter of largest IDTB	2.859(2.859,2.063)	0.000	1.569(1.162, 2.118)	0.003
Short diameter of largest IDTB	2.608(1.872,3.632)	0.000	-	-
Area of largest IDTB	3.434(2.084,5.659)	0.000	-	-
Myometrial thinning/disruption	2.065(1.460,2.919)	0.000	-	-
Increased placental vascularity	1.987(1.507,2.620)	0.000	-	-
Location of Increased placental vascularity	1.670(1.279,2.180)	0.000	13.804(2.839,67.114)	0.001
Diameter of Increased placental vascularity	2.413(1.696,3.434)	0.000	795.003(3.778,167270.939)	0.014
Placenta bulge	1.908(1.178,3.092)	0.009	-	-
Thickness of placenta	2.797(1.468,5.330)	0.002	-	-

**Histopathologic criteria model**				
	**Univariate logistic regression analyses**		**Multivariate logistic regression analyses**	
	**OR (95% CI)**	**P**	**OR (95% CI)**	**P**
Gestational age at CD , weeks	0.595(0.443,0.800)	0.001		
Number of VD	1.718(1.295,2.279)	0.000		
Number of CD	0.667(0.491,0.905)	0.009	0.603(0.364,0.999)	0.050
Type of placenta previa	0.654(0.485,0.882)	0.005		
Preoperative fibrinogen	0.590(0.441,0.790)	0.000	0.575(0.413,0.801)	0.001
Preoperative hemoglobin level	0.738(0.565,0.964)	0.026	1.695(1.140,2.521)	0.009
IDTB	2.201(1.671,2.899)	0.000		
Number of IDTB	3.019(2.056,4.432)	0.000		
Long diameter of largest IDTB	2.945(2.076,4.177)	0.000	1.615(1.176,2.217)	0.003
Short diameter of largest IDTB	2.762(1.914,3.987)	0.000		
Area of largest IDTB	3.409(2,030,5.726)	0.000		
Myometrial thinning/disruption	2.145(1.513,3.042)	0.000		
IPV	1.963(1.4787,2.607)	0.000		
Location of IPV	1.666(1.263,2.198)	0.000	7.689(1.795,32.933)	0.006
Diameter of IPV	2.425(1.677,3.507)	0.000		
Placenta bulge	2.145(1.302,3.534)	0.003		
Placenta cover uterus scar	1.962(1.481,2.599)	0.000		
Thickness of placenta	3.719(1.599,8.646)	0.002		

Note.CD=cesarean delivery, IDTB=intraplacental dark T2 bands, IPV=Increased placental vascularity, OR=Odds Ratio, VD=Vaginal delivery

#### Histopathologic criteria model constructing

##### Radiomics model

**Fig. 5:**
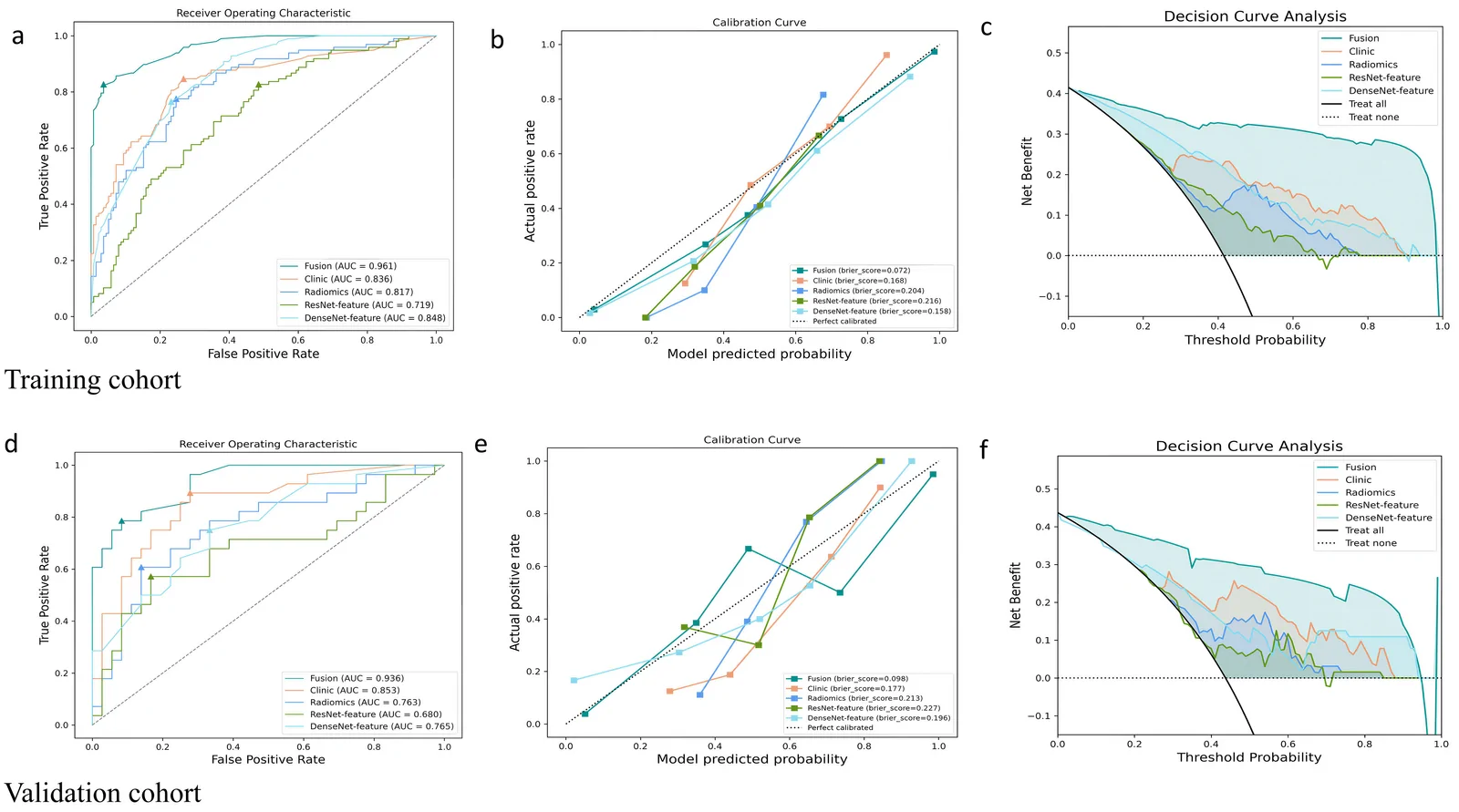
**a**. ROC curves of histopathological criteria model, including radiomics model, DenseNet deep learning model, ResNet deep learning model and fusion model in training cohort. **b**. Calibration curve of different models in training cohort. **c**. decision curve of different models in training cohort. **d**. ROC curves of histopathological criteria model, including radiomics model, DenseNet deep learning model, ResNet deep learning model and fusion model in validation cohort. **e**. decision curve of different models in validation cohort. **f**. decision curve of different models in validation cohort

The radiomics model based on 16 selected features yielded AUCs of 0.817 (95% CI:0.762,0.872) and 0.763 (95% CI: 0.641,0.885) in the training and validation cohorts, respectively. It also exhibited consistent classification metrics, with ACC, SN, and SP of 0.758, 0.765, 0.754 in the training cohort and 0.719, 0.714, 0.722 in the validation cohort (Table 2; Figs. 5a, d).

##### ResNet model

The AUC of ResNet in the training cohort was 0.719 (95% CI: 0.654,0.784), ACC of 0.657, SN of 0.694, and SP of 0.630, and in the validation cohort the AUC was 0.68 (95% CI: 0.539,0.820), ACC of 0.625, SN of 0.714, and SP of 0.556 (Table 2; Figs. 5a, d).

##### DenseNet model

The AUC of DenseNet deep learning model for constructing histopathologic criteria model was 0.848 (95% CI: 0.801,0.895) in the training cohort, 0.765 (95% CI: 0.648,0.882) in the validation cohort. Performance metrics were as follows: ACC, SN, and SP were 0.733, 0.898, and 0.616 in the training cohort; and 0.656, 0.786, and 0.556 in the validation cohort. (Table 2; Figs. 5a, d).

##### Clinical model

Using single factor binary logistic regression, 18 clinical features were screened in clinical model (Table 3). The AUC value of the clinic model constructed was 0.836 (95% CI: 0.782,0.890) in the training cohort. Its SN was 0.684, SP was 0.804, and ACC was 0.754. The AUC value of the clinical model constructed was 0.853 (95% CI: 0.759,0.948) in the validation cohort. Its SN was 0.857, SP was 0.722, and ACC was 0.781 (Table 2; Figs. 5a, d).

#### Model comparison and fusion

After comparing performance, the fusion models for both intraoperative clinical and histopathologic criteria-based PAS diagnosis significantly outperformed the individual models in both the training and validation cohorts. For the intraoperative clinical criteria model, the fusion model achieved an AUC of 0.964 (95% CI: 0.946, 0.982) in the training cohort and 0.949 (95% CI: 0.902, 0.996) in the validation cohort (Table 2; Figs. 4a, d). Their AUC values were 0.961 (95% CI 0.940–0.982) and 0.936 (95% CI 0.883–0.990) in the training and validation cohorts of histopathologic criteria model, respectively (Table 2; Figs. 5a, d).

Both fusion models (intraoperative clinical and histopathologic criteria-based) exhibited good calibration in the training and validation cohorts (Figs. 4b and e and 5b and e), with predicted probabilities closely aligning with observed outcomes. Furthermore, decision curve analysis (DCA) showed that the fusion models provided a higher net benefit across most threshold probabilities compared to all individual models (Figs. 4c and f and 5c and f), indicating superior clinical utility.

**Fig. 6:**
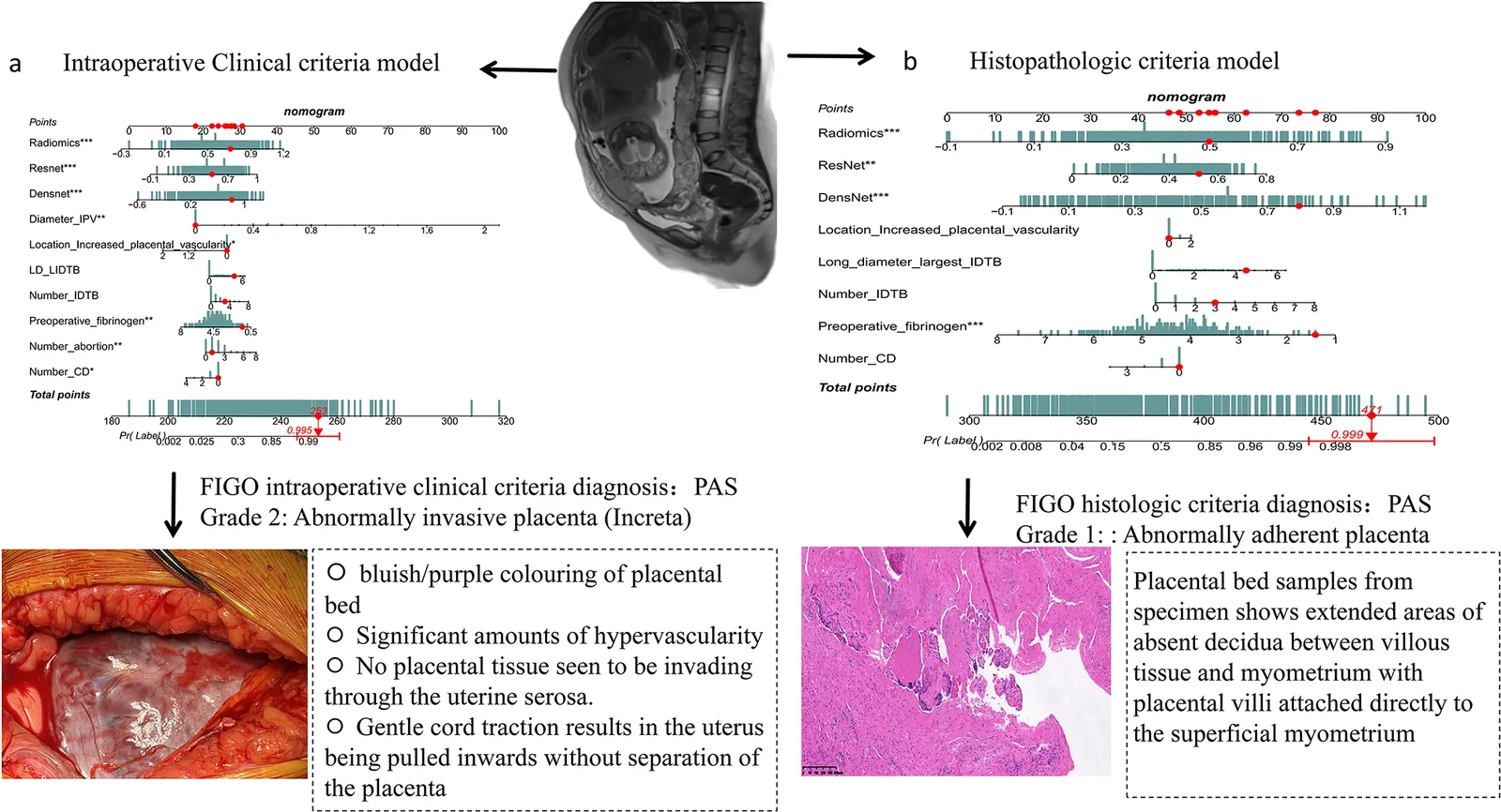
Nomogram of dual model for intraoperative clinical and histopathologic criteria diagnosis which showed an example of predicting the intraoperative clinical and pathological diagnosis of PAS in pregnant women suspected of having PAS before caesarean section, separately. **a**. Nomogram of intraoperative clinical diagnostic criteria model and intraoperative condition of the parturient. **b**. Nomogram of histopathologic diagnostic criteria model and pathological results and grading of postpartum women

Clinical indicators, Rad-score, ResNet-score, and DenseNet-score were used to construct dual diagnostic nomograms for **intraoperative** clinical and histopathologic criteria (Figs. 6a, b).

#### The correlation between clinical indicators, deep learning, and radiomics

**Fig. 7:**
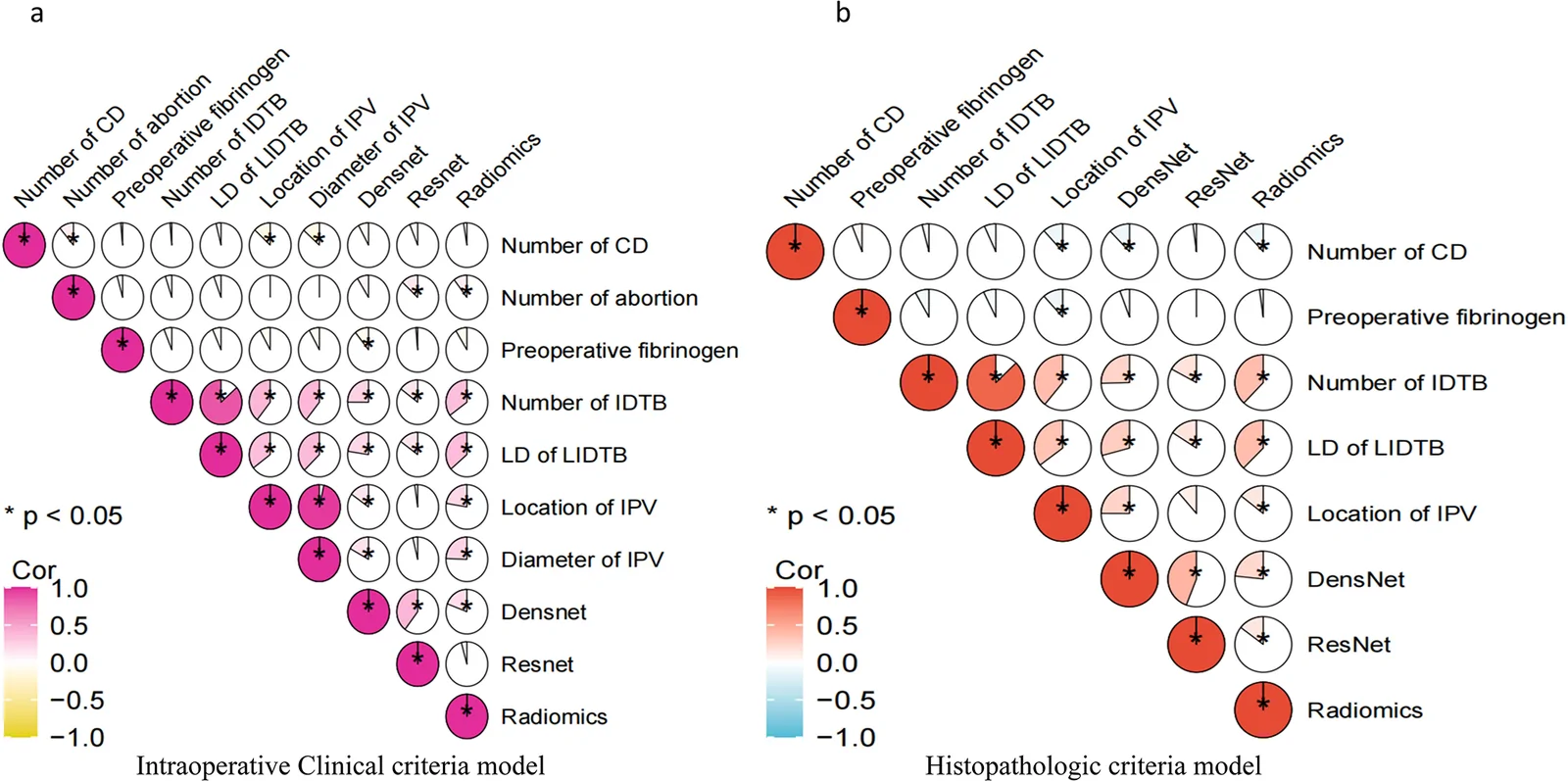
Correlation Pie Chart of matrix. **a**. correlation matrix of intraoperative clinical criteria model **b**. correlation matrix of histopathologic criteria model. Rows and columns represent variables. The filling ratio of each pie chart represents the correlation coefficient between variables. The darker the color, the more correlated the variables are. The marked * in the pie chart represents *p* < 0.05

Correlation matrices were used to visualize the relationships between clinical indicators, radiomic score, ResNet score, and DenseNet score in the nomograms of the **intraoperative** clinical and histopathologic criteria models, respectively (Figs. 7a, b). Correlation coefficients and P-values are provided in Supplementary Tables S3a, S3b, S4a, and S4b.We found that the count of intraplacental dark T2 bands (IDTB) (*r* = 0.35, *P* < 0.001; *r* = 0.381, *P* < 0.001) and the long diameter of the largest intraplacental dark T2 band (LIDTB) (*r* = 0.362, *P* < 0.001; *r* = 0.378, *P* < 0.001) exhibited moderate correlations with radiomics scores in the **intraoperative** clinical model and histopathological model, respectively. The scores derived from ResNet and DenseNet were also significantly correlated in the **intraoperative** clinical model (*r* = 0.396, *P* < 0.001) and histopathological model (*r* = 0.443, *P* < 0.001), respectively.

## Discussion

Our study grouped pregnancies suspected of PAS before cesarean section based on FIGO’s intraoperative clinical and histopathologic diagnostic criteria. Based on this grouping, artificial intelligence (AI) methods, including radiomics and deep learning, were used to develop dual fusion models based on intraoperative clinical and histopathologic criteria.

AI applications in medical imaging primarily include radiomics and deep learning. The main clinical applications of AI in PAS research include preoperative prediction of PAS [31], postpartum hemorrhage [21, 32] and placental invasion depth [33]. Numerous studies have developed AI models to predict PAS versus PAS-negative cases [34]. This is inaccurate for guiding clinical management.

In our study, we constructed a intraoperative clinical and histopathologic criteria dual model. Furthermore, our study integrated radiomics, deep learning, and clinical features to develop a fusion model. To address the limited predictive performance and suboptimal generalization of individual models, we employed ensemble learning. This approach leveraged the complementary strengths of each model, resulting in enhanced performance for PAS prediction. Additionally, based on previous quantitative studies on PAS signs, we included quantitative data related to LIDTB and IPV as clinical features [27, 28].

Notably, we developed a dual model with excellent performance and stability. The fusion model for the intraoperative clinical criteria achieved AUCs of 0.964 and 0.949 in the training and validation cohorts, respectively. Similarly, the fusion model for the histopathologic criteria achieved AUCs of 0.961 and 0.936 in the training and validation cohorts, respectively The performance of the dual fusion models in this study was validated using calibration and decision curves. Meanwhile, we also explored the correlations among radiomics scores, ResNet scores, DenseNet scores from deep learning, and key clinical indicators. Radiomics and deep learning are highly complementary in medical image analysis. Radiomics provides interpretable, handcrafted features, while Deep Learning offers powerful, automated feature extraction and modeling capabilities. Many AI studies on various diseases adopt the integration of these two methods [35, 36]. We conducted a correlation analysis between deep learning scores, radiomic scores and several clinical features. We found that radiomics features associated with grayscale values were extracted in both intraoperative clinical and histopathologic criteria models, such as original_firstorder_Maximum, NGTDM, GLSZM, GLRLM, GLCM and GLDM. The count of IDTB and the long diameter of the LIDTB represent the MR feature most closely correlated with grayscale. Pathologically, IDTB arise from obliterated villous architecture, erythrocyte extravasation and pyknosis of syncytiotrophoblast nuclei. The intervillous spaces become constricted and subsequently occupied by fibrin deposits. Fibrous tissues and vestigial villous remnants encircle old infarcts [27, 37]. IDTB have been widely recognized in numerous studies as key signs for diagnosing placenta accreta spectrum [38, 39]. Accordingly, these radiomic features are strongly correlated with the underlying pathophysiological changes in placenta. This also indicates that the model we developed is internally consistent and interpretable.

We acknowledge several limitations. Most notably, the sample size remains relatively limited despite our two-center design. To enhance model stability, we combined cases from both centers for model development, resulting in a lack of external validation from a third center. Second, the images analyzed in our study were limited to the sagittal HASTE sequence from Siemens scanners. In the future, multiple sequences will be included for research. Third, the evaluation of imaging features by radiologists might still be subjective.

Based on the FIGO diagnostic criteria for PAS, we constructed histopathologic clinical and histopathological criteria fusion models. This model fused artificial intelligence methods of radiomics, deep learning, and important clinical features. In the future, obstetricians might evaluate the intraoperative condition and pathology of suspected PAS pregnant women separately before caesarean section.

## Supplementary Information

Below is the link to the electronic supplementary material.

Supplementary Material 1

Supplementary Material 2

Supplementary Material 3

Supplementary Material 4

## Data Availability

Our study was a retrospective study, therefore there was no clinical registration. The datasets used and analyzed during the current study are available from the corresponding author on reasonable request.

## Abbreviations

PAS: Placenta accreta spectrum
MRI: Magnetic resonance imaging
US: Ultrasound
ESUR: European Society of Urogenital Radiology
SAR: Society of Abdominal Radiology
IDTB: Intraplacental dark T2 bands
IPV: Increased placental vascularity
LD: Long diameter
LIDTB: Largest intraplacental dark T2 band
CD: Cesarean Delivery
APTT: Activated partial thromboplastin time
CNN: Convolutional neural network
SVM: Support vector machine
