# Supplementary material for: Predicting placenta accreta spectrum antenatally: a deep learning-radiomics dual-model based on intraoperative clinical and histopathologic criteria
Source: BMC Med Imaging. 2026 Jul 17;26:520. doi: 10.1186/s12880-026-02538-2 (PMC13660289; doi:10.1186/s12880-026-02538-2)
Supplement: Supplementary file 1 [file 12880_2026_2538_MOESM1_ESM.docx]

**Figure S1:**Confusion matrices of PAS(+)or PAS(-) classification by the clinical,radiomics, DenseNet，ResNet and fusion models in the (A) training, (B) validation cohorts in histopathologic and intraoperative clinical criteria model. The color depends on the number inside the square: the higher the number, the darker the color.1 represents PAS (+), 0 represents PAS (-).
