# Supplementary material for: Predicting placenta accreta spectrum antenatally: a deep learning-radiomics dual-model based on intraoperative clinical and histopathologic criteria
Source: BMC Med Imaging. 2026 Jul 17;26:520. doi: 10.1186/s12880-026-02538-2 (PMC13660289; doi:10.1186/s12880-026-02538-2)
Supplement: Supplementary file 2 [file 12880_2026_2538_MOESM2_ESM.doc]

**Supplementary Methods**

**Appendix E1** Details of radiomics feature extraction and radiomics model development

The texture features consisted of 21 gray level cooccurrence matrix (GLCM) features, 14 gray level-dependent matrix (GLDM) features, 16 gray level run-length matrix (GLRLM) features, 16 gray level size zone matrix (GLSZM) features, and 5 neighborhood gray-tone difference matrix (NGTDM) features. Additionally, wavelet and LoG filters were applied to the original images, and the identical features were also derived from the filtered images. Consequently, a grand total of 1185 features were successfully extracted from each subject.

The values of radiomics features were normalized with Z-scores. The feature selection strategy is influenced by the stability of the features. To avoid the overfitting and reduce the computational complexity, we reduced the feature space dimension by the following three steps: To begin, the Mann–Whitney U test was used to measure the difference between each feature’s distribution within the PAS and non-PAS, and features with P > 0.05 were removed. Then, to eliminate redundant features, Pearson correlation coefficients between features were calculated, and feature pairs with correlation coefficients over 0.90 were eliminated one with a large P value in the previous step. Finally, features with non-zero coefficients were selected as the final features group using the least absolute shrinkage and selection operator (LASSO).

**Appendix E2** Details of training deep learning models

To optimize the parameters of deep learning models, the bounding box was defined as the bounding cube of the placenta region of interest (ROI). All ROI cubes were resized to a dimension of 96×96×96 via linear interpolation. To enrich the dataset and improve the model's generalization ability, data augmentation techniques were implemented. Specifically, X, Y, and Z axis inversion strategies were applied. Subsequently, the 3D images of the primary placenta, along with their corresponding placenta ROIs, were used as input for the 3D deep convolutional neural network (DCNN). The network parameters were updated through the backpropagation algorithm and optimized using the Adam optimizer. The training process spanned 300 epochs, with a learning rate set at 0.02.

During training, two losses were used to train our deep learning models: the classification loss and the extra attention loss for individual cases. We adopt the binary cross entropy as constrain for the PAS/non-PAS classification loss. For the individual patient, we can use the ROI of placenta to directly refine the attention maps from our model. Two kinds of data augmentation were applied during the training stage of deep learning to avoid overfitting. First, random cropping was implemented by randomly placing the segmented placenta image in the fixed cube with shape. Second, a fixed window center (WC) and window width (WW) of 300 were applied for input images with original CT values. A random change was applied for the training data with WW value ranging from −10 to 10 and WC value from −5 to 5. After successful training, the trained 3D DCNN was capable of predicting the PAS (presumably a specific metric related to the patient) for a given patient. To acquire the 3D deep learning (DL) features of an individual patient, a total of 256-d features were extracted from the penultimate fully connected layer of the DCNN models.
