# Supplementary material for: Predicting placenta accreta spectrum antenatally: a deep learning-radiomics dual-model based on intraoperative clinical and histopathologic criteria
Source: BMC Med Imaging. 2026 Jul 17;26:520. doi: 10.1186/s12880-026-02538-2 (PMC13660289; doi:10.1186/s12880-026-02538-2)
Supplement: Supplementary file 3 [file 12880_2026_2538_MOESM3_ESM.doc]

Supplemental table 1 MRI parameters of the two centers

| Hospital | Scanner | Patients No. | Sequence | TR/TE  (ms) | Slice Thickness (mm) | Slice Gap (mm) | Matrix size | FOV  (mm) |
| --- | --- | --- | --- | --- | --- | --- | --- | --- |
| Center 1 | SIEMENS 1.5T  (MAGENTOM Area) | 207 | Sagittal T2 | 1200/90 | 6 | 0 | 256×288 | 380×344 |
| Centre 2 | SIEMENS 1.5T  (MAGENTOM Avanto) | 194 | Sagittal T2 | 1350/90 | 6 | 0 | 256×205 | 380×380 |

Supplemental table 2 Radiological characteristics of patients in Intraoperative Clinical and Histopathologic criteria model

| Dual model | | Intraoperative Clinical criteria model | | | | | | | Histopathologic criteria model | | | | | | | | |  |
| --- | --- | --- | --- | --- | --- | --- | --- | --- | --- | --- | --- | --- | --- | --- | --- | --- | --- | --- |
|  | | Training cohort | | | Testing cohort | | | p-inter | Training cohort | | | | | Testing cohort | | | p-inter |  |
| Characteristics | | PAS-  (n=136) | PAS+  (n=179) | p-intra | PAS-  (n=35) | PAS+  (n=51) | p-intra |  | PAS-  (n=138) | PAS+  (n=98) | | | p-intra | PAS-  (n=36) | PAS+  (n=28) | p-intra |  |  |
| IDTB | -  + | 103 (75.735)  33 (24.265) | 74 (41.341)  105 (58.659) | p<.001* | 26 (74.286)  9 (25.714) | 17 (33.333)  34 (66.667) | p<.001* | 0.307 | 105 (76.087)  33 (23.913) | | 38 (38.776)  60 (61.224) | | p<.001* | 26 (72.222)  10 (27.778) | 5 (17.857)  23 (82.143) | p<.001* | 0.081 | |
| Number of IDTB | | 0.000 [0.000,1.000] | 1.000 [0.000,2.000] | p<.001* | 0.000 [0.000,0.500] | 1.000 [0.000,2.000] | p<.001* | 0.462 | 0.000 (0.000,1.000) | 1.000 (0.000,3.000) | | | p<.001* | 0.000 (0.000,1.000) | 2.000 (1.000,2.000) | p<.001* | 0.237 |  |
| Long diameter of largest IDTB | | 0.000 [0.000,0.000] | 1.600 [0.000,2.500] | p<.001* | 0.000 [0.000,0.200] | 1.700 [0.000,2.750] | p<.001* | 0.207 | 0.000 (0.000,0.000) | 1.600 (0.000,2.700) | | | p<.001* | 0.000 (0.000,0.600) | 2.100 (1.175,2.825) | p<.001* | 0.083 |  |
| Short diameter of largest IDTB | | 0.000 [0.000,0.000] | 0.700 [0.000,1.300] | p<.001* | 0.000 [0.000,0.100] | 0.900 [0.000,1.200] | p<.001* | 0.327 | 0.000 0.000,0.000) | 0.800 (0.000,1.475) | | | p<.001* | 0.000 (0.000,0.225) | 0.950 (0.775,1.225) | p<.001* | 0.114 |  |
| Area of largest IDTB | | 0.000 [0.000,0.000] | 1.320 [0.000,3.020] | p<.001* | 0.000 [0.000,0.060] | 1.530 [0.000,3.000] | p<.001* | 0.257 | 0.000 (0.000,0.000) | 1.645 (0.000,3.513) | | | p<.001* | 0.000 (0.000,0.226) | 1.955 (0.948,3.255) | p<.001* | 0.103 |  |
| Placenta bulge | -+ | 135 (99.265)  1 (0.735) | 161 (89.944)  18 (10.056) | 0.001* | 34 (97.143)  1 (2.857) | 48 (94.118)  3 (5.882) | 0.513 | 0.626 | 137 (99.275)  1 (0.725) | | 84 (85.714)  14 (14.286) | | p<.001* | 35 (97.222)  1 (2.778) | 25 (89.286)  3 (10.714) | 0.193 | 0.975 | |
| Myometrial thinning/disruption | -+ | 131 (96.324)  5 (3.676) | 139 (77.654)  40(22.347) | p<.001* | 33 (94.286)  2 (5.714) | 38 (74.510)  13 (25.490) | 0.018* | 0.601 | 133 (96.377)  5 (3.623) | | 73 (74.490)  25(25.510) | | p<.001* | 34 (94.444)  2 (5.556) | 19 (67.857)  9 (32.143) | 0.005* | 0.454 | |
| IPV | -  + | 123 (90.441)  13 (9.559) | 117 (65.363)  62 (34.637) | p<.001* | 32 (91.429)  3 (8.571) | 34 (66.667)  17 (33.333) | 0.008* | 0.915 | 125 (90.580)  13 (9.420) | | 63 (64.286)  35 (35.714) | | p<.001* | 32 (88.889)  4 (11.111) | 16 (57.143)  12 (42.857) | 0.004* | 0.419 | |
| Location of IPV | -  F  M | 123 (90.441)  2(1.471)  11(8.088) | 117 (65.363)  39 (21.788)  23 (12.849) | p<.001* | 32 (91.429)  1 (2.857)  2 (5.714) | 34 (66.667)  8 (15.686)  9 (17.647) | 0.03* | 0.839 | 124 (89.855)  2 (1.449)  12 (8.696) | | 61 (62.245)  23 (23.469)  14 (14.286) | | p<.001* | 32 (88.889)  2 (5.556)  2 (5.556) | 16 (57.143)  5 (17.857)  7 (25.000) | 0.016* | 0.753 | |
| Diameter of IPV | | 0.000 (0.000,0.000) | 0.000 (0.000,0.400) | p<.001* | 0.000 (0.000,0.000) | 0.000 (0.000,0.500) | 0.005* | 0.93 | 0.000 (0.000,0.000) | 0.000 (0.000,0.400) | | | p<.001* | 0.000 (0.000,0.000) | 0.000 (0.000,0.500) | 0.003* | 0.472 |  |
| Bladder wall interruption | -  + | 134 (98.529)  2 (1.471) | 172 (96.089)  7(3.911) | 0.473 | 35 (100.000)  0 (0.000) | 49 (96.078)  2 (3.922) | 0.236 | 1 | 136 (98.551)  2 (1.449) | | 94 (95.918)  4 (4.081) | | 0.264 | 36 (100.000)  0 (0.000) | 26 (92.857)  2 (7.143) | 0.103 | 0.72 | |
| Thickness of placenta | | 3.950 (3.300,4.800) | 4.300 (3.450,5.600) | 0.012* | 3.800 (3.000,4.500) | 4.100 (3.450,5.150) | 0.108 | 0.227 | 3.950 (3.300,4.800) | 4.350 (3.425,5.700) | | | 0.015* | 3.750 (3.000,4.500) | 4.400 (3.575,5.450) | 0.030* | 0.617 |  |
| Placenta cover uterus scar | -  + | 112 (82.353)  24 (17.647) | 98 (54.749)  81(45.251) | p<.001* | 28 (80.000)  7 (20.000) | 30 (58.824)  21 (41.177) | 0.076 | 1 | 114 (82.609)  24 (17.391) | | | 53 (54.082)  45 (45.918) | p<.001* | 28 (77.778)  8 (22.222) | 13 (46.429)  15 (50.571) | 0.017* | 0.484 | |

**Abbreviations: IDTB=intraplacental dark T2 bands,IPV=increased placental vascularity,F=fetal surface,M=maternal surface**

|  | Number of birth | Number of abortion | Preoperative fibrinogen | Number of IDTB | LD of LIDTB | Location of IPV | Diameter of IPV | Densnet | Resnet | Radiomics |
| --- | --- | --- | --- | --- | --- | --- | --- | --- | --- | --- |
| Number of birth | / | 0.112 | 0.015 | -0.0146 | -0.0381 | -0.131 | -0.134 | -0.0791 | -0.062 | -0.0252 |
| Number of abortion | 0.112 | / | -0.043 | 0.0498 | 0.0558 | 0.00842 | 0.00912 | 0.0884 | 0.13 | 0.104 |
| Preoperative fibrinogen | 0.015 | -0.043 | / | -0.0612 | -0.0656 | -0.0801 | -0.0798 | -0.108 | -0.0145 | -0.0878 |
| Number of IDTB | -0.0146 | 0.0498 | -0.0612 | / | 0.865 | 0.393 | 0.392 | 0.249 | 0.149 | 0.35 |
| LD of LIDTB | -0.0381 | 0.0558 | -0.0656 | 0.865 | / | 0.352 | 0.373 | 0.226 | 0.15 | 0.362 |
| Location of IPV | -0.131 | 0.00842 | -0.0801 | 0.393 | 0.352 | / | 0.966 | 0.153 | 0.023 | 0.224 |
| Diameter of IPV | -0.134 | 0.00912 | -0.0798 | 0.392 | 0.373 | 0.966 | / | 0.17 | 0.0381 | 0.245 |
| Densnet | -0.0791 | 0.0884 | -0.108 | 0.249 | 0.226 | 0.153 | 0.17 | / | 0.396 | 0.193 |
| Resnet | -0.062 | 0.13 | -0.0145 | 0.149 | 0.15 | 0.023 | 0.0381 | 0.396 | / | 0.043 |
| Radiomics | -0.0252 | 0.104 | -0.0878 | 0.35 | 0.362 | 0.224 | 0.245 | 0.193 | 0.043 | / |

Supplemental table 3a Correlation coefficient of Intraoperative clinical model

**Abbreviations: VD=Vaginal delivery,IDTB=intraplacental dark T2 bands,LIDTB=largest intraplacental dark T2 bands,IPV=increased placental vascularity,LD=long diameter**

Supplemental 3b P-value of correlation analysis of Intraoperative clinical models

|  | Number of birth | Number of abortion | Preoperative fibrinogen | Number of IDTB | LD of LIDTB | Location of IPV | Diameter of IPV | Densnet | Resnet | Radiomics |
| --- | --- | --- | --- | --- | --- | --- | --- | --- | --- | --- |
| Number of birth | / | 0.0243 | 0.765 | 0.77 | 0.447 | 0.00856 | 0.007 | 0.114 | 0.215 | 0.616 |
| Number of abortion | 0.0243 | / | 0.391 | 0.319 | 0.265 | 0.867 | 0.855 | 0.077 | 0.00915 | 0.0373 |
| Preoperative fibrinogen | 0.765 | 0.391 | / | 0.222 | 0.191 | 0.11 | 0.111 | 0.0311 | 0.773 | 0.0795 |
| Number of IDTB | 0.77 | 0.319 | 0.222 | / | 0.000 | 0.000 | 0.000 | 0.000 | 0.003 | 0.000 |
| LD of LIDTB | 0.447 | 0.265 | 0.191 | 0.000 | / | 0.000 | 0.000 | 0.000 | 0.003 | 0.000 |
| Location of IPV | 0.00856 | 0.867 | 0.11 | 0.000 | 0.000 | / | 0.000 | 0.002 | 0.646 | 0.000 |
| Diameter of IPV | 0.007 | 0.855 | 0.111 | 0.000 | 0.000 | 0.000 | / | 0.001 | 0.447 | 0.000 |
| Densnet | 0.114 | 0.077 | 0.0311 | 0.000 | 0.000 | 0.002 | 0.001 | / | 0.000 | 0.000 |
| Resnet | 0.215 | 0.00915 | 0.773 | 0.003 | 0.003 | 0.646 | 0.447 | 0.000 | / | 0.391 |
| Radiomics | 0.616 | 0.0373 | 0.0795 | 0.000 | 0.000 | 0.000 | 0.000 | 0.000 | 0.391 | / |

**Abbreviations: VD=Vaginal delivery,IDTB=intraplacental dark T2 bands,LIDTB=largest intraplacental dark T2 bands,IPV=Increased placental vascularity,LD=Long diameter**

Supplemental table 4a Correlation coefficient of histopathologic model

|  | Number of birth | Preoperative fibrinogen | Number of IDTB | LD of LIDTB | Location of IPV | DensNet | ResNet | Radiomics |
| --- | --- | --- | --- | --- | --- | --- | --- | --- |
| Number of birth | / | 0.0573 | -0.039 | -0.0659 | -0.115 | -0.119 | -0.0194 | -0.117 |
| Preoperative fibrinogen | 0.0573 | / | -0.0791 | -0.0707 | -0.114 | -0.0549 | -0.00606 | -0.0211 |
| Number of IDTB | -0.039 | -0.0791 | / | 0.875 | 0.392 | 0.254 | 0.169 | 0.381 |
| LD of LIDTB | -0.0659 | -0.0707 | 0.875 | / | 0.354 | 0.293 | 0.157 | 0.378 |
| Location of IPV | -0.115 | -0.114 | 0.392 | 0.354 | / | 0.25 | 0.111 | 0.142 |
| DensNet | -0.119 | -0.0549 | 0.254 | 0.293 | 0.25 | / | 0.443 | 0.233 |
| ResNet | -0.0194 | -0.00606 | 0.169 | 0.157 | 0.111 | 0.443 | / | 0.146 |
| Radiomics | -0.117 | -0.0211 | 0.381 | 0.378 | 0.142 | 0.233 | 0.146 | / |

**Abbreviations: VD=Vaginal delivery,IDTB=intraplacental dark T2 bands,LIDTB=largest intraplacental dark T2 bands,IPV=Increased placental vascularity,LD=Long diameter**

Supplemental 4b P-value of correlation analysis of histopathologic model

|  | Number of birth | Preoperative fibrinogen | Number of IDTB | LD of LIDTB | Location of IPV | DensNet | ResNet | Radiomics |
| --- | --- | --- | --- | --- | --- | --- | --- | --- |
| Number of VD | / | 0.323 | 0.501 | 0.256 | 0.047 | 0.039 | 0.738 | 0.043 |
| Preoperative fibrinogen | 0.323 | / | 0.173 | 0.223 | 0.048 | 0.344 | 0.917 | 0.717 |
| Number of IDTB | 0.501 | 0.173 | / | 0.000 | 0.000 | 0.000 | 0.003 | 0.000 |
| LD of LIDTB | 0.256 | 0.223 | 0.000 | / | 0.000 | 0.000 | 0.006 | 0.000 |
| Location of IPV | 0.047 | 0.048 | 0.000 | 0.000 | / | 0.000 | 0.055 | 0.014 |
| DensNet | 0.039 | 0.344 | 0.000 | 0.000 | 0.000 | / | 0.000 | 0.000 |
| ResNet | 0.738 | 0.917 | 0.003 | 0.006 | 0.055 | 0.000 | / | 0.012 |
| Radiomics | 0.043 | 0.717 | 0.000 | 0.000 | 0.014 | 0.000 | 0.012 | / |

**Abbreviations:VD=Vaginal delivery,IDTB=intraplacental dark T2 bands,LIDTB=largest intraplacental dark T2 bands,IPV=Increased placental vascularity,LD=Long diameter**
