# Supplementary material for: Predicting placenta accreta spectrum antenatally: a deep learning-radiomics dual-model based on intraoperative clinical and histopathologic criteria
Source: BMC Med Imaging. 2026 Jul 17;26:520. doi: 10.1186/s12880-026-02538-2 (PMC13660289; doi:10.1186/s12880-026-02538-2)
Supplement: Supplementary file 4 [file 12880_2026_2538_MOESM4_ESM.doc]

**The final selected feature subset of the histopathologic criteria model:**

**Deep Learning Deep Features:**

The majority of selected features are deep learning-derived features (n=28), including 20 DenseNet features (dense_dl_*) and 8 ResNet features (resnet_dl_*). This reflects the complementary feature extraction capabilities of DenseNet and ResNet, with DenseNet features contributing more prominently to capturing local fine-grained lesion patterns.

**Radiomics Features:**

Wavelet transform-based features: Texture features (wavelet_glcm_*, wavelet_ngtdm_*) and first-order statistical features (wavelet_firstorder_*), which capture multi-scale and multi-directional lesion information.

LoG filter-based features: Texture features (log_glcm_*) derived from Laplacian of Gaussian (LoG) filtered images.

**Shape feature:** original_shape_Flatness, a morphological feature extracted from original images.

**Clinical/Imaging Morphological Features:** 8 features related to typical PAS imaging and clinical manifestations, such as long_T2WI_strip_low_signal (T2WI hypointense strip), abnormal_vessel (abnormal vessels), cover_uterus_scar (placenta covering uterine scar), and discontinuous_myometrium (discontinuous myometrium).

**Obstetric/Demographic Features:** Variables including born_age (maternal age at delivery), num_birth (parity), type_placenta_previa (type of placenta previa), and length_cervical_canal (cervical canal length), reflecting the auxiliary predictive value of obstetric background information.

**Laboratory Marker:** preoperative_fibrinogen (preoperative fibrinogen level), the only laboratory indicator included in the final model.

**The final selected feature subset of the intraoperative clinical criteria model:**

**Deep Learning Deep Features:**

The largest group of selected features are deep learning-derived features (n=24), including 17 DenseNet features (dense_dl_*) and 7 ResNet features (resnet_dl_*). This highlights the complementary roles of DenseNet and ResNet in capturing PAS-related imaging patterns, with DenseNet features contributing more to local fine-grained lesion characterization.

**Radiomics Features:**

Wavelet transform-based features: Encompass first-order statistical features (e.g., wavelet_firstorder_wavelet-LHL-Skewness, wavelet_firstorder_wavelet-HLH-Mean) and texture features (e.g., wavelet_glcm_*, wavelet_glszm_*, wavelet_ngtdm_Busyness), capturing multi-scale and multi-directional lesion heterogeneity.

Laplacian of Gaussian (LoG) filter-based features: Include first-order statistical features (e.g., log_firstorder_log-sigma-0-5-mm-3D-Kurtosis) and texture features (e.g., log_glcm_wavelet-LLH-GrayLevelNonUniformityNormalized), enhancing edge and lesion boundary detection.

**Original image-based features:** original_ngtdm_Busyness (texture) and original_firstorder_Maximum (first-order statistics), derived from unfiltered images.

**Clinical/Imaging Morphological Features:** 5 features representing typical PAS imaging and clinical history, including long_T2WI_strip_low_signal (T2WI hypointense strip), diameter_abnormal_vessel (abnormal vessel diameter), abnormal_vessel (abnormal vessels), num_abortion (number of abortions), and cover_uterus_scar (placenta covering uterine scar).

**Laboratory Marker:** preoperative_fibrinogen (preoperative fibrinogen level), the only laboratory indicator retained in the final clinical model.
